# Durlobactam in combination with β-lactams to combat *Mycobacterium abscessus*

**DOI:** 10.1128/aac.01174-24

**Published:** 2024-12-23

**Authors:** Eunjeong Shin, Khalid M. Dousa, Magdalena A. Taracila, Christopher R. Bethel, Mary Nantongo, David C. Nguyen, Chidiebere Akusobi, Sebastian G. Kurz, Mark S. Plummer, Charles L. Daley, Steven M. Holland, Eric J. Rubin, Jürgen B. Bulitta, W. Henry Boom, Barry N. Kreiswirth, Robert A. Bonomo

**Affiliations:** 1 Louis Stokes Cleveland VA Medical Center 2546 , Cleveland, Ohio, USA; 2 Department of Medicine, Case Western Reserve University School of Medicine 12304 , Cleveland, Ohio, USA; 3 Department of Molecular Biology and Microbiology, Case Western Reserve University School of Medicine 12304 , Cleveland, Ohio, USA; 4 Division of Infectious Diseases, Department of Pediatrics and Division of Infectious Diseases, and Department of Internal Medicine, Rush University Medical Center 2468 , Chicago, Illinois, USA; 5 Department of Immunology and Infectious Diseases, Harvard T. H. Chan School of Public Health 1857 , Boston, Massachusetts, USA; 6 School of Medicine, Yale University 12228 , New Haven, Connecticut, USA; 7 Biopharmaworks, Groton, Connecticut, USA; 8 Division of Mycobacterial and Respiratory Infections, National Jewish Health 2930 , Denver, Colorado, USA; 9 Laboratory of Clinical Immunology and Microbiology, National Institutes of Health, National Institute of Allergy and Infectious Diseases 35037 , Bethesda, Maryland, USA; 10 Department of Pharmacotherapy and Translational Research, College of Pharmacy, University of Florida 686060 , Orlando, Florida, USA; 11 Center for Discovery and Innovation, Hackensack Meridian Health, Nutley, New Jersey, USA; 12 CWRU-Cleveland VAMC Center for Antibiotic Resistance and Epidemiology (Case VA CARES), Cleveland, Ohio, USA; 13 Departments of Biochemistry, Pharmacology, and Proteomics and Bioinformatics, Case Western Reserve University 2546 , Cleveland, Ohio, USA; 14 Department of Pharmacology, School of Medicine, Case Western Reserve University 2546 , Cleveland, Ohio, USA; 15 Department of Proteomics and Bioinformatics, Case Western Reserve University School of Medicine 12304 , Cleveland, Ohio, USA; 16 Cleveland Geriatric Research Education and Clinical Center (GRECC), VANEOHS 273136 , Cleveland, Ohio, USA; Johns Hopkins University School of Medicine, Baltimore, Maryland, USA

**Keywords:** *Mycobacterium abscessus*, synergistic bacterial killing, beta-lactams, beta-lactamase inhibitors

## Abstract

*Mycobacterium abscessus* (*Mab*) presents significant clinical challenges. This study evaluated the synergistic effects of a β-lactam and β-lactamase inhibitor combination against *Mab* and explored the underlying mechanisms. Synergy was assessed through MIC tests and time-kill studies, and binding affinities of nine β-lactams and BLIs to eight target receptors (L,D-transpeptidases [LDT] 1–5, D,D-carboxypeptidase, penicillin-binding protein [PBP] B, and PBP-lipo) were assessed using mass spectrometry and kinetic studies. Thermal stability and morphological changes were determined. Imipenem demonstrated high binding affinity to LDTs and PBPs, with extremely low inhibition constants (*K_i_*_,*app*_; ≤0.002 mg/L for LDT1−2, ≤0.6 mg/L for PBPs), while cephalosporins, sulopenem, tebipenem, and amoxicillin exhibited moderate to low binding affinity. Durlobactam inactivated Bla_Mab_ and LDT/PBPs more potently than avibactam. The *K_i,app_*s of durlobactam for PBP B, PBP-lipo, and LDT2 were below clinically achievable unbound concentrations, while avibactam’s *K_i,app_* for LDT/PBPs exceeded the clinical concentrations. Single β-lactam treatments resulted in minimal killing (~1 log_10_ reduction). Although avibactam yielded no effect, combinations with avibactam showed a significant reduction (~4 log_10_ CFU/mL). Durlobactam alone showed ~2 log_10_ reduction, and when combined with imipenem or two β-lactams, durlobactam achieved near-eradication of *Mab*, surpassing the current therapy (amikacin + clarithromycin + imipenem/cefoxitin). Inactivation of PBP-lipo by sulopenem, imipenem, durlobactam, and amoxicillin (with avibactam) led to morphological changes, showing filaments. This study demonstrates the mechanistic basis of combinations therapy, particularly imipenem + durlobactam, in overcoming β-lactam resistance in *Mab*.

## INTRODUCTION

*Mycobacterium abscessus* (*Mab*), a non-tuberculous mycobacterium (NTM), presents significant challenges to clinicians (1) due to its intrinsic resistance to first-line anti-mycobacterial drugs and the majority of currently available antibiotics. *Mab* infections often result in chronic pulmonary disease, particularly in patients with immunosuppression or structural lung diseases such as bronchiectasis and cystic fibrosis, and are associated with high mortality rates (2). *Mab* infections in cystic fibrosis patients are notoriously difficult to treat, with failure rates of 50%–75%, often leading to a rapid decline in lung function (3, 4). The presence of macrolide resistance genes (*erm41* and *rrl*) in the *Mab* subspecies, whether induced or acquired, can contribute to high treatment failure rates (~70%) (5, 6), surpassing those observed with multidrug-resistant tuberculosis and even extensively drug-resistant tuberculosis (7, 8).

Treating *Mab* infections poses significant challenges, requiring prolonged therapy with multidrug regimens, which could result in a high risk of toxicity. Current guidelines for combating *Mab* recommend at least three antimicrobial regimens, including aminoglycoside amikacin (9). However, treatment outcomes for *Mab* infections remain poor compared to those with other mycobacteria (10). Furthermore, amikacin is associated with significant otic and renal toxicity (11–13).

β-Lactams are well established for their safety across all age groups and are therefore well-suited for long-term combination therapy involving multiple β-lactams. In NTMs, β-lactams exert their antibacterial effect by inhibiting both L,D-transpeptidases (LDTs) and D,D-transpeptidases (DDTs, also known as penicillin-binding protein; PBPs) (14). These enzymes catalyze the final crosslinking step of peptidoglycan biosynthesis. While PBPs form transpeptide linkages between the fourth and third residues of peptide side chains (4→3 linkages), LDTs, which are predominant in *Mab*, form alternative 3→3 linkages (15). Although LDTs are the primary drivers of peptidoglycan biosynthesis in *Mab*, the inhibition of PBPs such as PBP-lipo (*MAB_3167*c), DDC, PBP B (*MAB_2000*), and DacB1 remains crucial for preventing the growth of or killing of *Mab*, as previously reported (16–18). Inactivating LDTs and PBPs interferes with the synthesis, maturation, and remodeling of the bacterial cell wall, which ultimately leads to bacterial cell death. Unfortunately, PBP and LDT binding data for β-lactams and BLIs in *Mab* are largely lacking, with only a few published reports (19, 20).

During the past two decades, substantial efforts in the development of new β-lactamase inhibitors (BLIs) have led to the introduction of several new drugs, including avibactam, relebactam, vaborbactam, and durlobactam (21). These BLIs have been utilized to combat multidrug-resistant Gram-negative bacteria. However, significantly less effort has been dedicated to evaluating their effects on *Mab* and rationally selecting the appropriate partner β-lactams, largely due to the paucity of mechanistic insights into *Mab* β-lactam resistance. *Mab* expresses a class A β-lactamase enzyme, Bla_Mab_ (22), which actively hydrolyzes penicillins and cephalosporins, although penems (including carbapenems and thiopenems) appear to be more resistant to hydrolysis by Bla_Mab_ (20, 22, 23). Despite the presence of Bla_Mab_, current treatment guidelines do not address this issue as well as do not include any BLIs in recommendations (9). Recent studies have observed that the newly developed DBO class of BLIs, including avibactam, nacubactam, zidebactam, and durlobactam, exhibit activity against Bla_Mab_ (23–29). However, the mechanisms of action for BLIs such as avibactam and durlobactam against *Mab* have not been thoroughly investigated.

We have previously demonstrated the activity of avibactam, relebactam, and durlobactam against Bla_Mab_ through kinetic and biochemical studies (20, 23). However, this does not fully elucidate the synergistic effects observed when BLIs are combined with β-lactams against *Mab*. BLIs inhibit β-lactamases, thereby protecting penicillins and cephalosporins from hydrolysis. Although carbapenems are not extensively hydrolyzed well by Bla_Mab_, the addition of avibactam or durlobactam showed a synergistic effect in *in vitro* studies (20, 23). This highlights a substantial mechanistic gap as no systematic studies have evaluated the bacterial responses to the combination of BLIs and β-lactams to achieve extensive killing of *Mab*.

This study aimed to evaluate the synergistic effects of β-lactam (BL) and β-lactamase inhibitor combinations against *Mab* and discover the underlying mechanisms of action by linking bacterial killing effects with target protein affinity (biochemical rationale). We hypothesized that durlobactam would function as “dual-action” compounds by enhancing the activity of β-lactam partners through protection from Bla_Mab_-mediated hydrolysis and by inhibition of target PBPs and LDTs, embodying the concept of “target redundancy.” To gain mechanistic insights into the actions of BL/BLI, we characterized the binding affinities of β-lactams to LDTs and PBPs. Conformational changes in the target enzymes upon β-lactam binding were examined using circular dichroism spectroscopy and differential scanning fluorimetry thermal shift assays. In addition, morphological changes in response to the inactivation of PBP-lipo were characterized using flow cytometry and automated confocal microscopy to gain insights into bacterial cell damage.

## RESULTS

### The MICs of *Mycobacterium abscessus* subsp. *abscessus* are lowered with the addition of BLI

To assess the synergistic effect of avibactam or durlobactam, these inhibitors were combined with a single β-lactam (sulopenem, imipenem; tebipenem, cefuroxime, ceftaroline, and amoxicillin) or dual β-lactams. While avibactam was tested at a fixed concentration of 4 mg/L, durlobactam + sulbacam were tested with β-lactams at a 1:1:1 ratio (i.e., durlobactam, sulbactam and the test β-lactam all at the same concentration) or a fixed concentration of 1 mg/L (i.e., durlobactam 1 mg/L + sulbactam 1 mg/L).

The MICs of imipenem, sulopenem, and cefuroxime showed no or slight reductions (approximately twofold decrease) upon the addition of avibactam (4 mg/L) or durlobactam +sulbactam (1 + 1 mg/L) (Table 1); this result may be consistent with previous observations showing these antibiotics are less hydrolyzed by Bla_Mab_ (20, 25). However, tebipenem, ceftaroline, and amoxicillin exhibited 4-fold to 64-fold MIC reductions with the addition of avibactam or durlobactam + sulbactam. The addition of avibactam or durlobactam + sulbactam to sulopenem + amoxicillin dramatically decreased the MICs of sulopenem (0.125 µg/mL or ≤0.0625 µg/mL; fourfold or ≥ eightfold reduction). Interestingly for the other combinations of two β-lactams (sulopenem + cefuroxime and imipenem + ceftaroline), the effects of avibactam and durlobactam + sulbactam on the MIC were diminished, showing a ≥1-fold reduction for sulopenem + cefuroxime, and a onefold or twofold reduction for imipenem + ceftaroline upon the addition of avibactam or durlobactam + SUL (Table 1).

**TABLE 1:** MICs were determined against *Mab* ATCC 19977 strain for the following drugs: durlobactam (DUR), sulbactam (SUL), avibactam (AVI), sulopenem (SULO), cefuroxime (CXM), amoxicillin (AMX), imipenem (IPM), ceftaroline (CFT), and tebipenem (TBP)^*a*^

β-lactam antibiotics	Alone	+ DUR + SUL (1:1)	+ DUR + SUL (1 mg/L)	+ AVI (4 mg/L)
DUR + SUL (1:1 ratio)	8	.	.	.
AVI	>128	.	.	.
SULO	1	0.25	1	0.5
CXM	4	1	2	4
AMX	128	1	1	32
IPM	8	2	4	4
CFT	16	.	2	1–2
TBP	8	1	1	2
SULO + CXM (2 mg/L)	≤0.0625	≤0.0625	≤0.0625	≤0.0625
SULO + CXM (4 mg/L)	≤0.0625	≤0.0625	≤0.0625	≤0.0625
SULO + AMX (4 µg/m)	0.5	≤0.0625	≤0.0625	0.125
IPM + CFT (1 µg/m)	0.25	.	0.125	0.25
*SOC treatment*				
Clarithromycin (CLM)	8			
Cefoxitin (FOX)	16			
Imipenem (IPM)	8			
Amikacin (AMK)	16			
AMK + CLM (0.4 mg/L)	32			
AMK + CLM (0.2 mg/L) +FOX (4 mg/L)	16			
AMK + CLM (0.2 mg/L) +IPM (1 mg/L)	16			

^*a*^ In addition, standard of care (SOC) treatments including clarithromycin (CLM), amikacin (AMK), cefoxitin (FOX), and imipenem (IPM) were tested in monotherapy, double, and triple combinations. The SOC triple combinations of AMK + CLM + FOX or AMK + CLM + IPM did not decrease the MIC compared to monotherapy.

The current standard-of-care (SOC) guidelines recommend at least a three-antibiotic combination including amikacin. However, combinations of amikacin, clarithromycin, and either cefoxitin or imipenem did not show MIC reduction, implying no synergistic effect and highlighting the need for optimal antibiotic combinations.

### BL/BLI exhibited a synergistic effect on bacterial killing in *in vitro* time-kill assays, as confirmed by Classification and Regression Tree analysis

**Fig 1:**
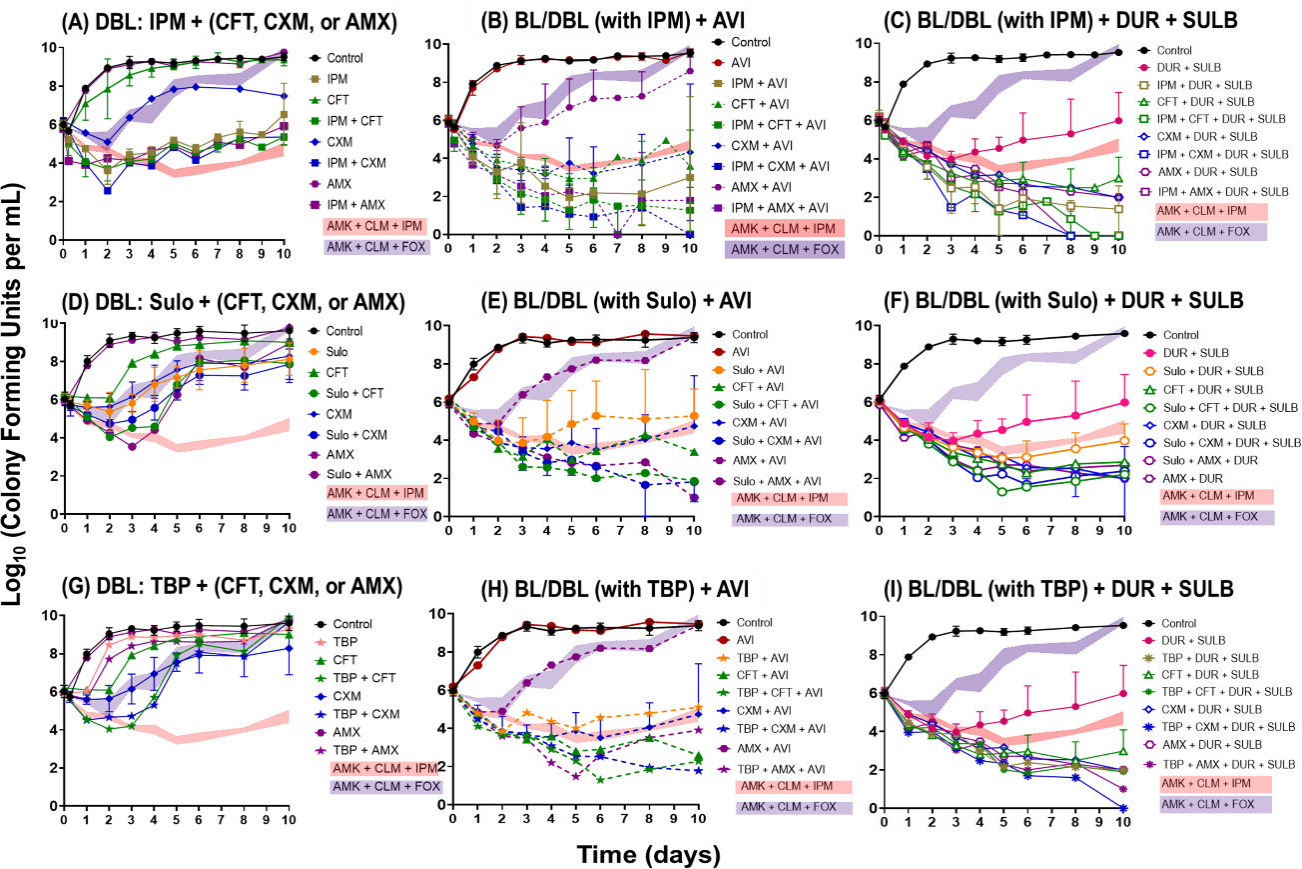
Time-kill curves were generated for single β-lactams: imipenem (IPM) (**A**), sulopenem (SULO) (**D**), and tebipenem (TBP) (**G**), as well as their combinations with either ceftaroline (CFT), cefuroxime (CXM), or amoxicillin (AMX) in the absence of β-lactamase inhibitors. Panels **B**, **E**, and **H** show a single or combination of two β-lactams in the presence of 4 mg/L avibactam (AVI). Panels **C**, **F**, and **I** represent single or dual β-lactams in the presence of 12 mg/L durlobactam (DUR) and 7 mg/L sulbactam (SUL). The SOC combinations of amikacin (AMK) + clarithromycin (CLM) + IPM (shown in pink band) or cefoxitin (FOX) (shown in purple band) were included for comparison of the killing effects. The concentrations of each drug used in this assay were clinically achievable: IPM at 12 mg/L, CFT at 8 mg/L, CXM at 8 mg/L, AMX at 2 mg/L, SULO at 2 mg/L, TBP at 1.5 mg/L, AMK at 12 mg/L, CLM at 0.3 mg/L, and FOX at 7 mg/L. To counteract thermal degradation, the following supplementation was performed every 48 h: 75% for IPM, 33% for SULO, 54% for TBP, 50% for CXM, and 55% for FOX.

We tested the antibacterial activity of single β-lactams and dual β-lactams by combining carbapenems (imipenem (Fig. 1A through C), sulopenem (Fig. 1D through F), or tebipenem (Fig. 1G through I)) with either cephalosporins (ceftaroline or cefuroxime) or a penicillin (amoxicillin), with or without BLIs (avibactam or durlobactam + sulbactam) (Fig. 1). Without BLIs, single β-lactams exhibited minimal antibacterial effects, with amoxicillin showing no killing effect and others achieving up to a 1 log_10_ CFU/mL reduction, with complete regrowth. Dual β-lactams resulted in more extensive bacterial killing, with ~2.5 log_10_ CFU/mL reduction. Dual β-lactams regimens including sulopenem (Fig. 1D) or tebipenem (Fig. 1G) demonstrated bacterial regrowth after 4 days, whereas those including imipenem (Fig. 1A) prevented regrowth over 10 days, achieving bacterial killing comparable to one of the SOC regimens (amikacin + clarithromycin + imipenem) and surpassing the efficacy of amikacin + clarithromycin + cefoxitin.

Notably, the addition of avibactam significantly decreased bacterial burden, resulting in a 3–4 log_10_ reduction, despite avibactam alone having no effect on bacterial growth inhibition or killing. When combined with avibactam, imipenem exhibited extensive killing among the tested β-lactams (Fig. 1B). Dual β-lactams + avibactam combinations surpassed the bacterial killing of single β-lactam + avibactam combinations (Fig. 1B, E, and H). Durlobactam emerged as the most effective BLI, enhancing bacterial killing and achieving near eradication (4–5 log_10_ reduction) over 10 days (Fig. 1C, F, and I. Similar to the effect observed with avibactam, when durlobactam was combined with single β-lactams, imipenem demonstrated the most extensive bacterial killing (Fig. 1C).

**Fig 2:**
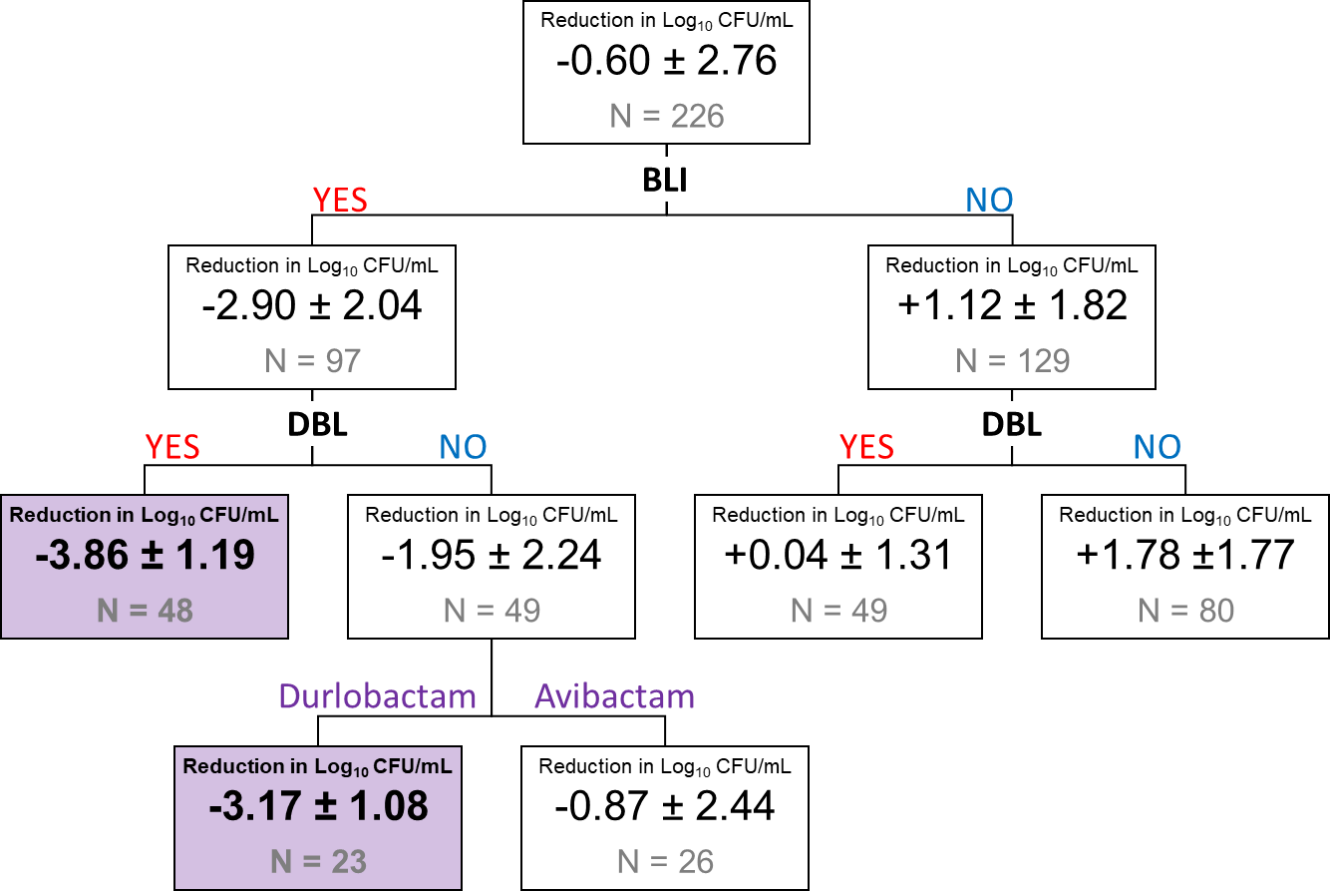
A classification tree was constructed based on bacterial burden reduction in log_10_ CFU/mL to determine the relative importance of predictor variables in the CART analysis. This CART analysis was performed with time-kill data collected on day 5 after dosing. Each split in the tree represents different regimens, such as β-lactamase inhibitors (BLIs), dual β-lactams (DBL), or BLI types. Data are average ± SD on the log_10_ scale, along with the number of treatment (or control) arms for various drug combinations in each category.

Our Classification and Regression Tree (CART) analysis revealed the most efficacious regimens for bacterial killing (Fig. 2). Without BLI, a dual β-lactams regimen was necessary to achieve bacterial stasis at 5 h. The addition of BLIs to dual β-lactams regimens resulted in the most extensive bacterial reduction (−3.86 ± 1.19 log_10_ CFU/mL). Among BL/BLI, combinations with durlobactam demonstrated the most extensive activity, achieving a −3.17 ± 1.08 log_10_ CFU/mL reduction compared to those with avibactam.

### β-Lactams and BLIs bind to peptidoglycan synthesizing proteins as well as Bla_Mab_

#### Mass spectrometry captured covalent adduct formation

**Fig 3:**
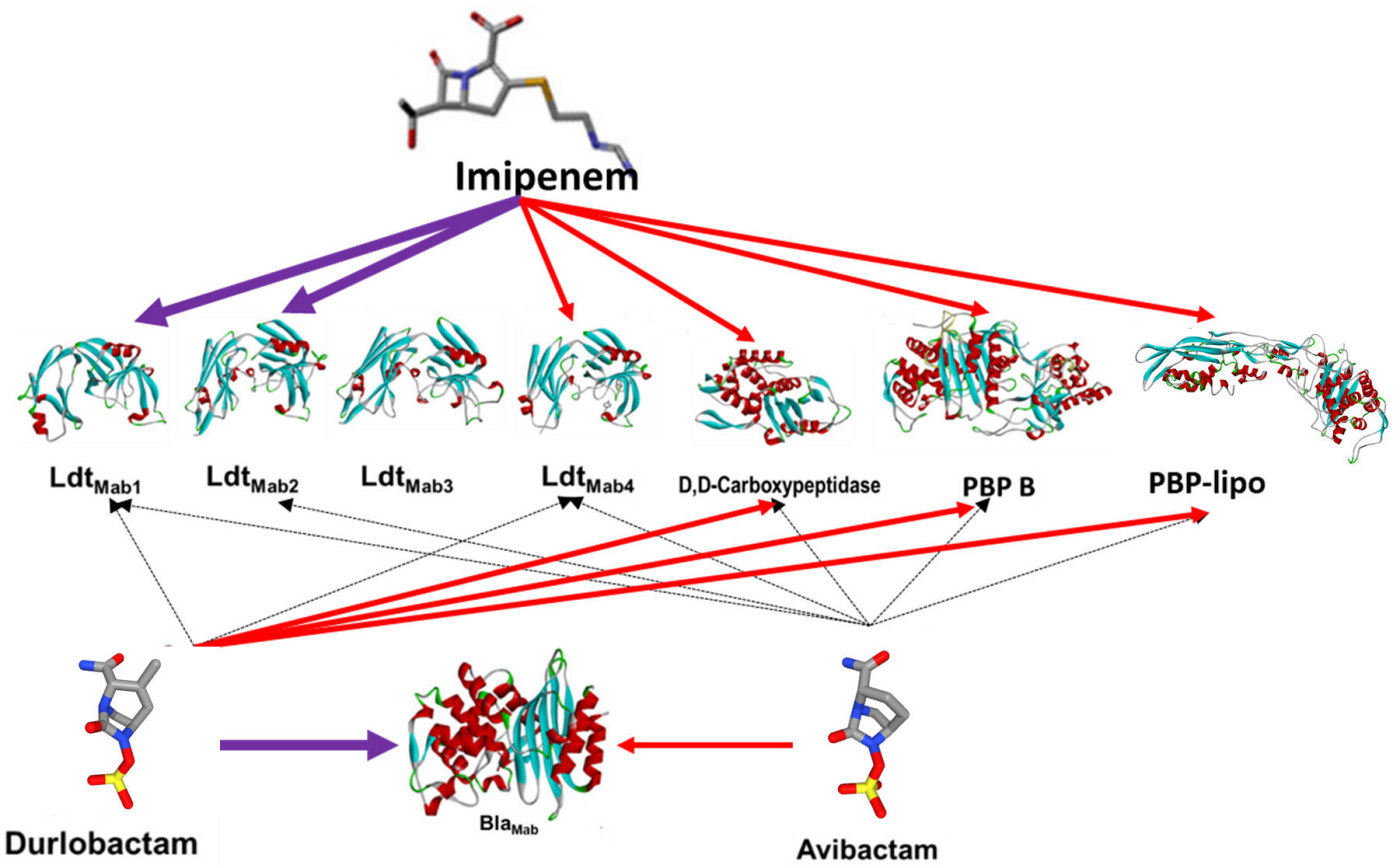
Redundancy and interaction between β-lactams or β-lactamase inhibitors (imipenem, avibactam, and durlobactam) and LDTs 1–4, D,D-carboxypeptidase, PBP B, PBP-lipo, and *Bla_Mab_*.

**Fig 4:**
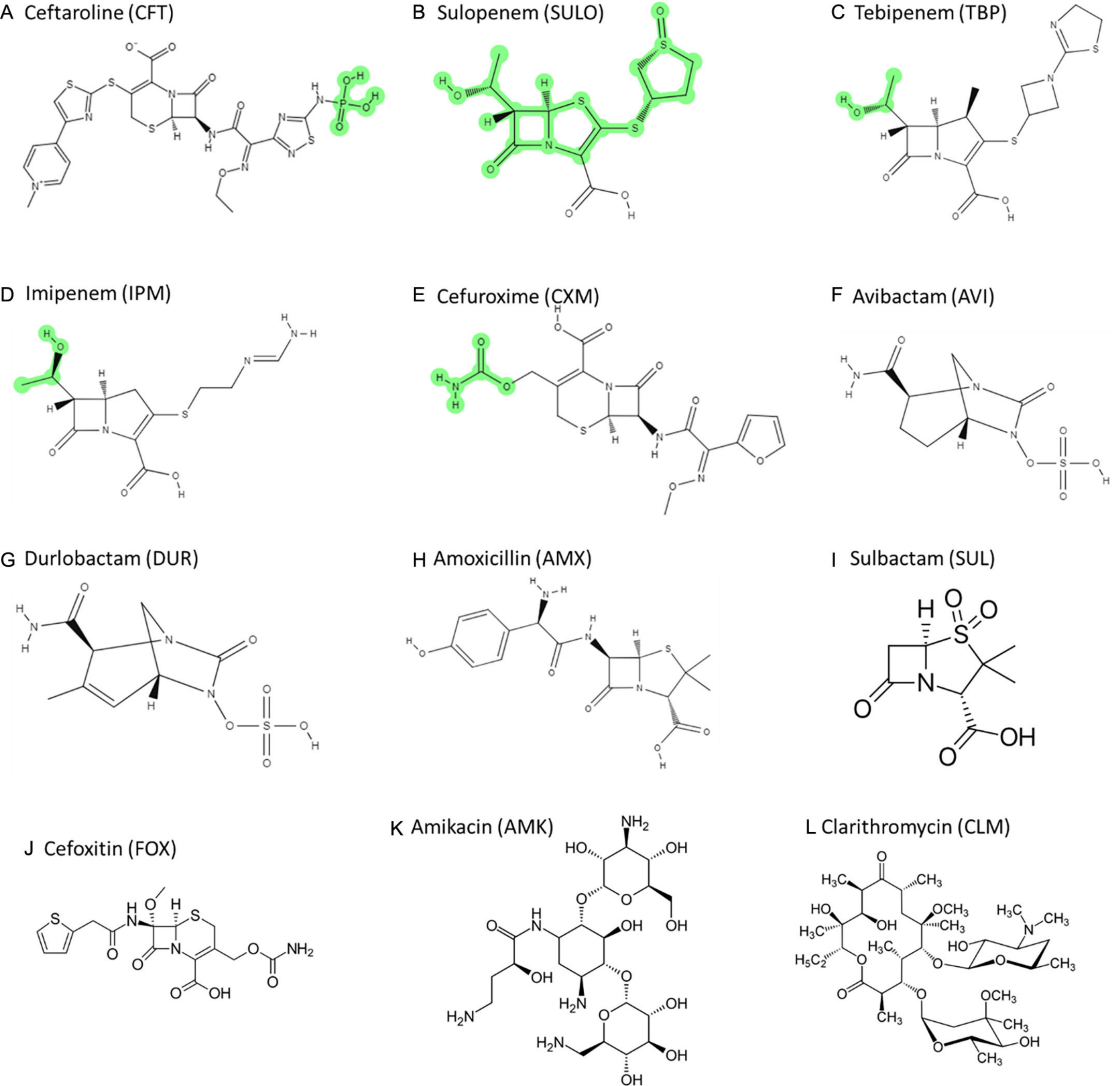
Chemical structures of β-lactams and β-lactamase inhibitors (**A**) ceftaroline (CFT); (**B**) sulopenem (SULO); (**C**) tebipenem (TBP); (**D**) imipenem (IPM); (**E**) cefuroxime (CXM); (**F**) avibactam (AVI); (**G**) durlobactam (DUR); (**H**) amoxicillin (AMX); (**I**) sulbactam (SUL); (**J**) cefoxitin (FOX) as well as the chemical structures of (**K**) amikacin and (**L**) clarithromycin. When ceftaroline, sulopenem, tebipenem, imipenem, and cefuroxime bind to LDTs or PBPs, they bind either as whole compounds or as fragments (Table 2). The portions expected to be removed upon binding to proteins are highlighted in green.

To confirm the inhibition of LDTs and PBPs by β-lactams and BLIs through covalent binding, we aimed to capture acyl-enzyme complexes between β-lactams/BLIs and target peptidoglycan enzymes (LDTs and PBPs). Previous studies have documented that some β-lactams and BLIs exhibited binding interaction with specific LDTs (20, 23, 25). In this study, we included PBP B and evaluated all tested β-lactams and BLIs (Table 2; Fig. 3). Durlobactam formed carbamoyl-enzyme complexes with all LDTs, except LDT3, as well as with PBP B and DDC. Imipenem and ceftaroline formed acyl-enzyme complexes with those. Avibactam also generated adducts with most LDTs (LDT1, LDT2, and LDT4), as well as with PBP B and DDC. Amoxicillin did not form acyl-enzyme complexes with most LDTs (LDT1-4) but did form complexes with PBPs (PBP B and DDC). Sulopenem formed acyl-enzyme complexes with PBP B, LDT1, LDT2, and LDT5. Tebipenem formed carbamoyl-enzyme complexes with PBP B, DDC, LDT1, LDT2, and LDT4. Ceftaroline, sulopenem, tebipenem, imipenem, and cefuroxime interact with LDTs or PBPs, binding either as complete molecules or as fragments (Fig. 4)

**TABLE 2:** Mass spectrometry analyses of LDTs 1–5, DDC, and PBP B conducted alone and in the presence of β-lactams and β-lactamase inhibitors. The mass accuracy was ±5 Da

	Observed MW (+ change in mw)						
	LDT1 (23,059)	LDT2 (39,216)	LDT3 (44,795)	LDT4 (32,567)	LDT5 (28,220)	DDC (26,788)	PBP B (62,915)
Avibactam (265)	23,325 (+265)	39,482 (+266)	44,794 (−)	32,566 (−) 32,831 (+265)	28,220 (−)	26,791 (−) 27,057 (+265)	63,180 (+265)
Durlobactam (277)	23,336 (+277)	39,492 (+278)	44,794 (−)	32,841 (+276)	28,496 (+276)	27,066 (+276)	63192 (+277)
Imipenem (299)	23,359 (+300) 23,316 (+257)	39,515 (+299)	44,794 (−)	32,866 (+299) 32,824 (+257)	28,476 (+256) 28,520 (+300)	27,090 (+299) 27,046 (+255)	63,215 (+300)
Sulopenem (349)	23,059 (−)	39,298 (+86)	45,143 (+349)	32,652 (+86)	28,306 (+86)	27,138 (+349)	63,264 (+349)
Tebipenem (384)	23,443 (+384)	39,602 (+386)	44,794 (−)	32,951 (+384)	28,220 (−)	27,173 (+385)	63,246 (+331) 63,297 (+382)
Ceftaroline (685)	23,744 (+685) 23,664 (+605)	39,901 (+685) 39,821 (+605)	44,794 (−)	33,252 (+685) 33,171 (+604)	28,220 (−) 28,825 (+605) 28,905 (+685)	27,396 (+605) 26,790 (−)	63,600 (+685) 63,520 (+605)
Cefuroxime (424)	23,059 (−)	39,597 (+381)	44,794 (−)	32,567 (−)	28,584 (+364)	28,584 (+364)	63,279 (+364)
Amoxicillin (365)	28,220 (−)	39, 214 (−)	44,794 (−)	32,841 (−)	28,220 (−)	27,156 (+366)	63,280 (+365)

These results highlight the capacity of different β-lactams and BLIs to bind to various LDTs and PBPs, thereby confirming their mechanism of action through covalent binding to these essential bacterial enzymes.

#### PBP-lipo binding was captured *via* Bocillin-FL

**Fig 5:**
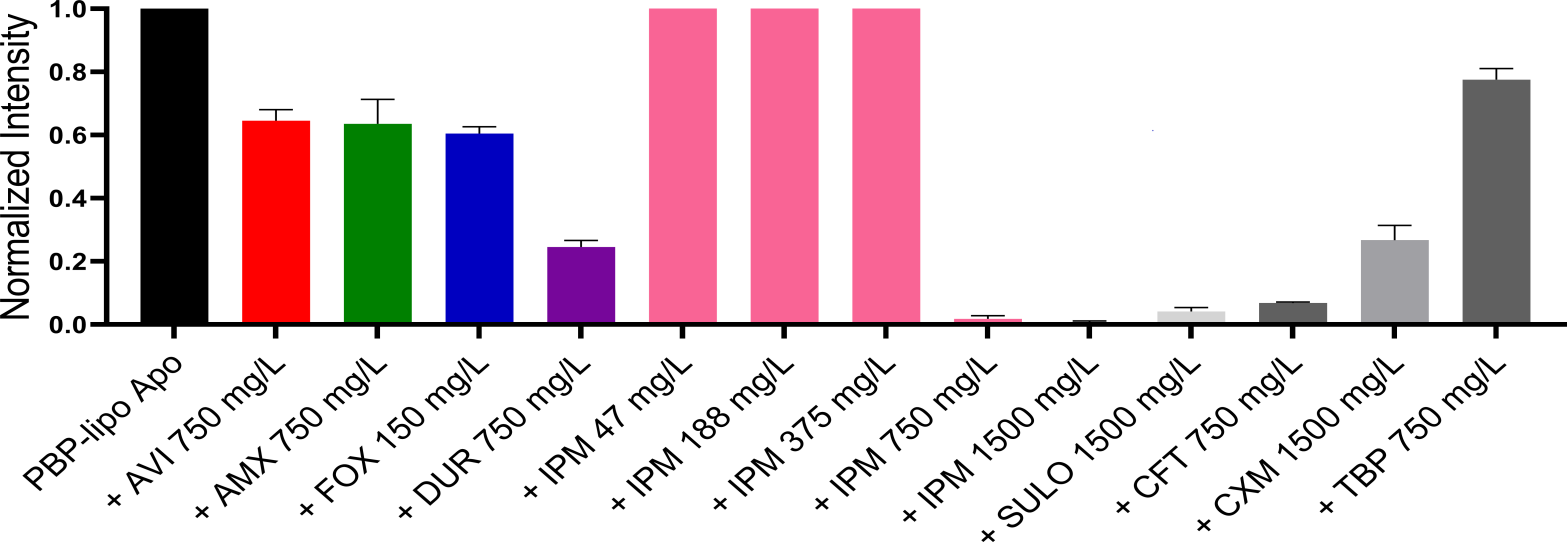
Binding of avibactam (AVI), amoxicillin (AMX), cefoxitin (FOX), durlobactam (DUR), imipenem (IPM), sulopenem (Sulo), ceftaroline (CFT), and cefuroxime (CXM) with PBP-lipo. The intensities of the PBP-lipo complex bands were quantified using GelAnalyzer software and normalized to the intensity of PBP-lipo without β-lactams (PBP-lipo Apo), which was set to 1. The low intensity of the PBP-lipo and Bocillin complex indicates that β-lactams are strongly bound to PBP-lipo.

Despite multiple attempts, we were unable to capture the PBP-lipo apo or the acyl complexes using mass spectrometry (MS). We hypothesized that this failure was due to the excessively long lipid chain of PBP-lipo, which might be incompatible with our MS system. To further investigate PBP-lipo activity, we employed the Bocillin FL assay, a commonly used method for identifying proteins with PBP activity. We incubated PBP-lipo with Bocillin-FL for 30 to 60 min but were unable to detect the PBP-lipo-Bocillin-FL complex (Fig. S1). This result aligned with previous reports indicating that PBP-lipo is a poor substrate for Bocillin-FL (as reported by Galanis et al. (16)). However, after extending the incubation period to over 3 h, we successfully detected the PBP-lipo-Bocillin-FL complex band (Fig. S1). Using the Bocillin-FL method, we then evaluated whether various β-lactams and BLIs could bind to PBP-lipo after 2 h of incubation (Fig. 5). Most of the tested β-lactams and BLIs, including durlobactam, avibactam, imipenem, ceftaroline, amoxicillin, sulopenem, and cefuroxime, demonstrated binding to PBP-lipo.

### Morphological changes

**Fig 6:**
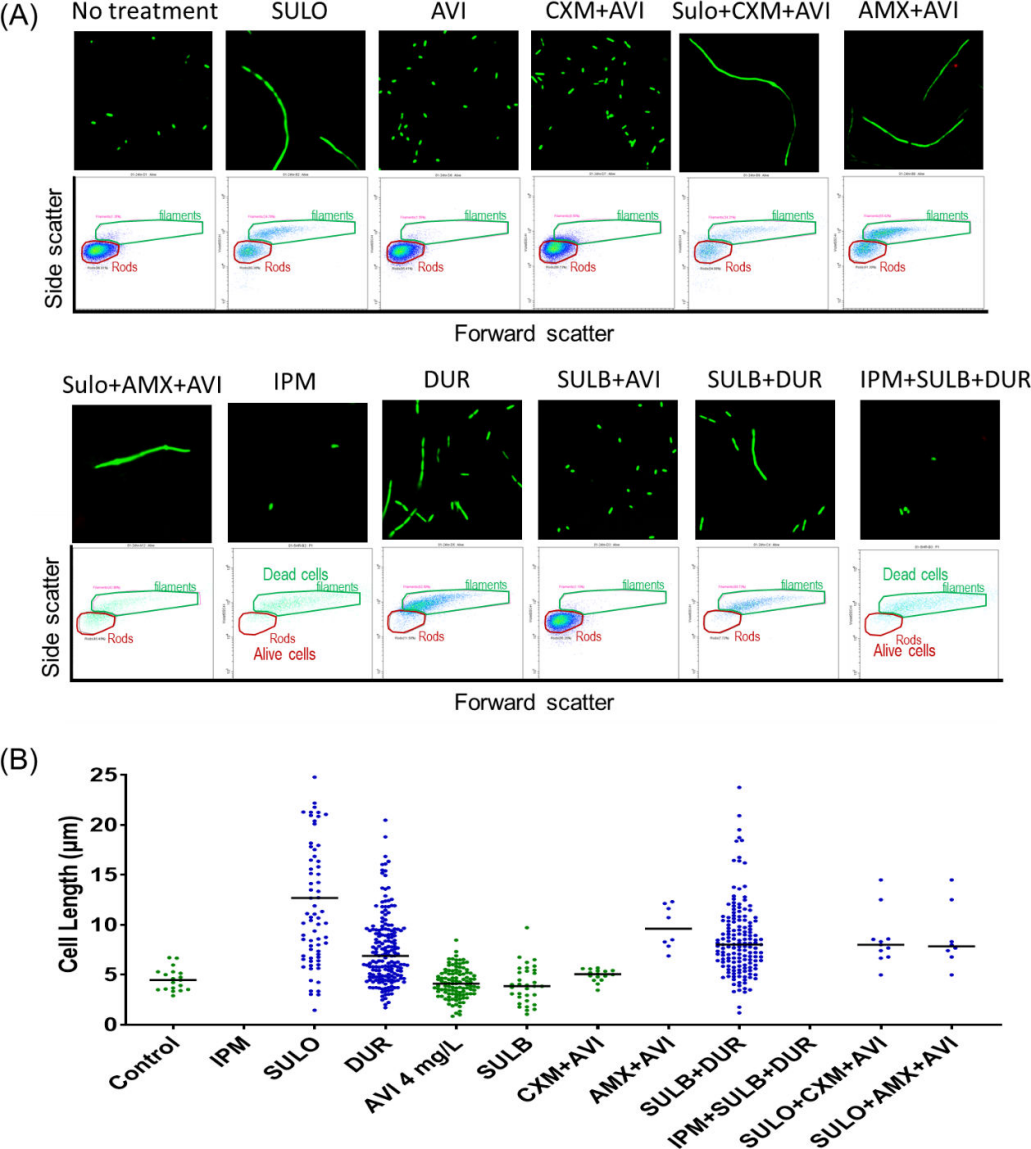
Confocal microscopy and flow cytometry assay, characterizing morphology changes in the *Mycobacterium abscessus* ATCC 19977 strain. Microscope image (×60) and forward scatter vs side scatter plot (**A**) exhibited that inactivation of PBP-lipo by sulopenem (SULO), amoxicillin (AMX) with avibactam (AVI), imipenem (IPM), or durlobactam (DUR) led to filamentous cells with significant bacterial killing at 24 h. (**B**) Cell length was determined through confocal microscopy image analysis. The lengths of all cells in the microscope sample at 24 h were measured, and the line represents the median value.

The morphological changes in response to PBP-lipo inhibition were assessed using flow cytometry and confocal microscopy. Rubin’s group previously demonstrated that a PBP-lipo knockdown strain of *Mab* exhibited disrupted cell morphology, characterized by the formation of multiple septa and elongated cell length (18). In this study, we explored the alterations in *Mab* cell morphology induced by β-lactams treatment targeting PBP-lipo (Fig. 6). In the absence of β-lactams, *Mab* cells maintained their typical rod shape. However, when treated with β-lactams and BLIs at clinically relevant unbound concentrations (i.e., the free average concentration at steady state, *f*C_ss,ave_), significant morphological changes were observed. Treatment with durlobactam, imipenem, amoxicillin + avibactam, and sulopenem changed *Mab* cells into filamentous shapes with an elongated cell length. Avibactam must be included with amoxicillin, as amoxicillin is highly hydrolyzed by Bla_Mab_ and does not induce any morphological changes by itself due to hydrolysis. Due to the rapid bacterial killing by imipenem, filamentous cells were predominantly detected in the dead cell gate during flow cytometry analysis. By contrast, other β-lactam and BLIs such as avibactam, cefuroxime, and sulbactam did not induce noticeable morphological changes, and *Mab* cells retained their rod shape.

### Kinetics of LDTs/PBPs by β-lactams and BLIs

We have investigated the binding profiles of β-lactams and BLIs to identify which LDTs and/or PBPs are targeted by these compounds through MS analysis and Bocillin-FL assay. Despite these findings, interpreting and linking these binding profiles to the bacterial killing effect remains challenging. To further understand the binding affinities of β-lactams and BLIs to LDTs and PBPs, we performed steady-state kinetic binding experiments using nitrocefin (NCF), a chromogenic cephalosporin substrate used to determine if a protein can hydrolyze the β-lactam ring.

Our results indicate that, although the BLIs, durlobactam and avibactam target most PBPs and LDTs, their preferred targets and binding affinities (i.e., inactivation constant, *K_i,app_*) differ significantly (Table 3). Durlobactam binds to PBP B, PBP-lipo, LDT2, and LDT5 with high (<1 mg/L) to mid (≤*f*C_ss,ave_) affinity. The *K_i,app_* values for these targets were lower than the clinically sustainable *f*C_ss,avg_, suggesting that durlobactam can effectively bind to these targets at clinically attainable concentrations. By contrast, *K_i,app_* of avibactam for PBP B, PBP-lipo, LDT1, and LDT2 were lower than *f*C_ss,ave_, indicating that these targets are unlikely to be significantly inhibited by avibactam in clinical settings.

**TABLE 3:** *K_i,app_* values of durlobactam (DUR), avibactam (AVI), imipenem (IPM), ceftaroline (CFT), amoxicillin (AMX), sulopenem (SULO), cefuroxime (CXM), sulbactam (SUL), and tebipenem (TBP) were determined for LDTs 1–5, PBP B, PBP-lipo, DDC, and *Bla_Mab_^a^*

*f*C_ss,avg_ (mg/L)^*b*^	16	5	12	7	6	2	12	7.5	1.7
K_*i,app*_ (mg/L)	DUR	AVI	IPM	CFT	AMX	SULO	CXM	SUL	TBP
*Penicillin-binding proteins*									
PBP B	16 (1)	9 (0.6)	0.4 (27)	1.4 (5)	2.7 (2)	2.9 (0.7)	3 (4)	13 (0.6)	0.007 (243)
PBP-lipo	0.1 (133)	5 (1)	0.6 (22)	14 (0.5)	0.1 (60)	0.08 (25)	22 (0.5)	.	34 (0.05)
DDC	10 (1.6)	37 (0.1)	1.3 (9)	23 (0.3)	0.04 (150)	21 (0.1)	0.05 (240)	31 (0.2)	127 (0.01)
*L,D-transpeptidases*									
LDT1	116 (0.1)	7 (0.7)	0.002 (5714)	0.5 (14)	.	.	.	150 (0.05)	1.9 (0.9)
LDT2	0.0002 (8000)	0.45 (11)	0.002 (6667)	0.7 (10)	.	0.07 (29)	0.5 (24)	0.02 (375)	0.01 (170)
LDT3	.	.	.	.	.	4.1 (0.4)	.	.	.
LDT4	2.7 (6)	>256 (<0.02)	4 (3)	68 (0.1)	.	1.7 (1.1)	.	3 (2.5)	0.7 (2.4)
LDT5	0.1 (145)	.	30 (0.4)	68 (0.1)	5 (1.2)	1.4 (1.5)	1.6 (7.5)	9 (0.8)	.
*BlaMab*	0.0011	0.0796	5.1	412	<5	.	.		<5

^*a*^ In addition, to assess whether binding could occur in clinical settings, clinically achievable concentrations (*f*C_ss_) were calculated and included. The ratio of FCss versus Kiapp was in parenthesis to compare. The higher value of the ratio than 1 means a higher possibility of hitting and the lower value means hard to hit those PBPs/LDTs in clinical settings. The ratio of *f*C_ss,avg_ to K_*i,app*_ is shown in parentheses for comparison. A ratio greater than 1 indicates a higher likelihood of targeting, while a ratio less than 1 suggests difficulty in targeting those PBPs/LDTs in clinical settings.

^*b*^ *f*C_ss,avg_ represents the median unbound average steady-state plasma concentration at clinically relevant doses. The predicted *f*C_ss,avg_ values of β-lactams and BLIs at clinically relevant doses are provided as continuous infusions. The *f*C_ss,avg_ for DUR was predicted at a dosage of 4,000 mg daily, for AVI at 1,500 mg daily, for IPM at 4,000 mg daily, for CFT at 1,800 mg daily, for AMX at 1,500 mg daily, for SULO at 1,000 mg daily, for CXM at 1,500 mg daily, for SUL at 4,000 mg daily, and for TBP at 1,800 mg daily. In the case of AMX, SULO, CXM, and TBP, which are available as oral agents as well, the *f*C_ss,avg_ would be lower than that of intravenous administration (see the supplemental material).

Among the β-lactams studied, imipenem showed extremely high affinity for LDT1 and LDT2, as well as high affinity for DDC, PBP B, and PBP-lipo, evidenced by low *K_i,app_* values (Table 3). This correlates with imipenem and imipenem-containing combination therapies yielding the most extensive bacterial killing of *Mab*. Other (carba)-penems, such as sulopenem and tebipenem, along with cephalosporins like ceftaroline and cefuroxime, also exhibited moderate binding to LDTs and PBPs. Amoxicillin showed moderate inhibition of PBP B, PBP-lipo, DDC, and LDT5. Sulbactam yielded no bacterial killing. With the exception of LDT2, sulbactam displayed poor binding affinity or no binding to most PBPs and LDTs, showing higher concentration compared to *f*C_ss,ave_, which required supra-physiological concentrations for binding.

### Differential scanning fluorimetry thermal shift binding assay

To gain further biochemical insights and understand the stabilizing effects of β-lactams and BLIs on LDTs and PBPs, we performed a differential scanning fluorimetry thermal shift assay. This assay was conducted in the presence and absence of the ligands (i.e., β-lactams and BLIs) to measure changes in protein melting temperatures (*Tm*). In the presence of β-lactams and BLIs targeting LDTs, we observed a decrease in *Tm* of the LDTs, indicating reduced thermal stability (Table 4; Fig. S2). Only ceftaroline binding to LDT1 resulted in a 1.5°C increase in *Tm*, suggesting a unique stabilizing effect. Both durlobactam and imipenem caused significant decreases in *Tm* of LDTs, with durlobactam showing the most substantial change. These results suggested that ligand binding generally decreased the thermal stability of LDTs, with the exception of ceftaroline which increased the thermal stability of LDT1.

**TABLE 4:** Melting temperatures (*Tm*, SD = ±0.2°C) of LDTs 1–5, DDC, PBP B, and PBP-lipo, and the changes in melting temperature in response to the binding of β-lactams and β-lactamase inhibitors

	LDT1 *Tm = 46.6*°C	LDT2 *Tm = 44.3*°C	LDT3 *Tm = 41.5*°C	LDT4 *Tm = 45.8*°C	LDT5 *Tm = 37.3*°C	DDC *Tm = 35.5*°C	PBPB *Tm = 31.6*°C	PBP-lipo *Tm = 42.4*°C
	ΔTm [°C]							
Imipenem	−5.8	−3.26	.	−2.6	−5.3	5.6	−3.4	−0.1
Sulopenem	.	−1.6	−1.1		0.9	10.4	1	
Ceftaroline	1.5	−0.77	.	-3	−1.4	−0.7	2.6	0.4
Cefuroxime	.	−0.4	.	.	−0.5	0.8	−2.6	
Amoxicillin	.	.	.	.	−1.3	2.8	7.6	-2
Sulbactam	−3.1	-3	.		0.5	0.8	−0.2	
Avibactam	−5.9	−2.6	.		.	4.2	−0.2	
Durlobactam	−9.1	−4.4	.		−1.7	0.4	−0.4	
IPM + CFT	−5.6	−1.97	.	−1.8	−6.1	5.4	−3.4	0
IPM + AMX	−6.8	−3.91	.			6.7		
CFT + AMX	1.8	−1.31	.		−0.7	1.1		

In contrast to LDTs, *Tm* of PBPs (DDC, PBP B, and PBP-lipo) generally increased in the presence of ligands targeting these proteins, indicating enhanced thermal stability. The binding of imipenem, sulopenem, amoxicillin, and avibactam to DDC resulted in increased *Tm* of the protein. Other β-lactams and BLIs binding to DDC caused less than 1°C change in *Tm*, indicating minimal or no impact on thermal stability. Imipenem and cefuroxime binding to PBP B decreased *Tm*, while sulopenem, ceftaroline, and amoxicillin increased the melting temperature. Interestingly, sulbactam, avibactam, and durlobactam, which bound PBP B with lower affinity, did not significantly change *Tm* (less than 0.5°C change). In terms of PBP-lipo, even though most of β-lactams and BLIs bound to this protein, only amoxicillin resulted in a decrease in *Tm* by 2°C, suggesting a distinct destabilizing effect.

## DISCUSSION

*Mab* infections are notoriously difficult to eradicate, exhibiting resistance to first-line antimycobacterial drugs as well as to many recently developed antibiotics (1). Despite this challenge, clinicians continue to use empiric, non-optimized antibiotic combinations to treat *Mab* infections. The current drug regimens pose significant safety concerns, and their efficacy remains questionable as previously reported (30, 31) and as shown by data presented in this study (in the SOC section of Table 1). A thorough understanding of the mechanisms of antibiotic action allows us to rationally optimize treatment regimens against *Mab*.

β-Lactams, the most extensively utilized and class of broad-spectrum antibiotics, have a well-documented history of effectively treating a broad range of bacteria (32). In recent years, there has been renewed interest in the repurposing and reintroduction of β-lactams as potential treatments for *Mab* infections. Currently, cefoxitin and imipenem are included in guideline recommendations as potential therapeutic options. However, numerous instances exist where *Mab* isolates exhibit resistance to one or both of these drugs (33). In addition, comprehensive LDTs/PBPs binding data of β-lactams in *Mab* are limited, with only a few studies reported (19, 20, 23). Understanding these interactions is essential for developing effective β-lactam-based combination therapies for *Mab* infections. Moreover, despite *Mab*’s resistance to most β-lactams, studies on BLIs and their significance have been relatively underemphasized. Herein, our work explored and expanded the number of β-lactams that demonstrate efficacy against *Mab*. This study demonstrated the synergistic bacterial killing effect of BL/BLI combinations against *Mab in vitro*. In addition, this analysis is the first to elucidate the underlying targets of BL/BLI combinations using a series of mechanistic and phenotypic assays in *Mab*.

Imipenem and sulopenem are not or very slowly hydrolyzed by Bla_Mab_ (20, 22, 23). Fortunately, combining imipenem or sulopenem with BLIs like avibactam or durlobactam resulted in significantly enhanced bacterial killing compared to imipenem or sulopenem alone (Fig. 1). We first assessed the binding affinities of BLIs to LDTs and PBPs (Table 3). Our results showed that durlobactam inhibited PBP-lipo, LDT2, and LDT5 with potent affinity and PBP B with mid-affinity. Most importantly, the clinically achievable unbound concentrations of durlobactam were higher than durlobactam *K_i,app_* for these targets (Table 3), indicating the potential for effective inhibition in clinical settings. In addition, durlobactam inhibits Bla_Mab_ more efficiently compared to other BLIs, such as avibactam or relebactam (24). By contrast, although avibactam inhibited PBPs and LDTs, its binding affinity was poor, and the *K_i,app_* values were higher than the unbound clinically achievable concentrations. This suggested that avibactam is unlikely to effectively bind to PBPs and LDTs under clinical conditions. Our biochemical studies are consistent with the results from MIC tests and time-kill studies. Durlobactam alone demonstrated significant killing of *Mab*, whereas avibactam did not exhibit any effect. These findings support our hypothesis that BLIs could have a dual action: inhibiting Bla_Mab_ and directly targeting PBPs and LDTs. This dual mechanism of action underscores the potential of BLIs, particularly durlobactam, as effective components of combination therapies against *Mab* infections.

The synergistic effects of the combinations imipenem + avibactam and sulopenem + avibactam (Fig. 1; Table 1) are not fully understood. Our kinetic study of avibactam indicates that avibactam cannot effectively inhibit LDTs and PBPs at 4 mg/L. We propose the following possible explanations for the observed synergy. (i) Mutation of Bla_Mab_. During treatment, mutations in Bla_Mab_ could occur, potentially leading to the hydrolysis of imipenem and sulopenem. By the addition of avibactam, imipenem and sulopenem were protected by hydrolysis by mutated Bla_Mab_. (ii) Target redundancy. Although avibactam alone does not inhibit LDTs and PBPs, it might enhance the inhibition of these targets when combined with β-lactams. Given that mutations are very rare and the ATCC 19977 strain only produces Bla_Mab_, we expect that avibactam may help inhibit LDTs and PBPs when combined with β-lactams. In addition, the synergistic killing effect of imipenem+avibactam and sulopenem+avibactam can also be attributed to avibactam’s inhibition of Bla_Mab_ (24).

While differential scanning fluorimetry analysis does not provide direct measurements of binding affinity for protein/ligand pairs (34), it offers insights into changes in protein stability. Consequently, a clear correlation between protein stability changes and binding affinity, arising from combined enthalpic and entropic effects, may not be evident (35). Nevertheless, our differential scanning fluorimetry thermal shift assay data suggest that the binding of β-lactams and BLIs to LDTs and PBPs differentially affected their thermal stability, reflecting diverse biochemical interactions and stabilizing effects. Our findings show that while β-lactams bind to specific LDTs or PBPs, not all exhibited significant changes in melting temperature. For instance, although it is evident that ceftaroline covalently bound to LDT2 and altered its secondary structure, as confirmed by our MS assay and circular dichroism spectroscopy (Fig. S3), the change in melting temperature was less than 1°C, suggesting minimal or no thermal stability alteration. In addition, most β-lactams bound to PBP-lipo with varying affinities, but expected changes in melting temperature were not observed (Table 4).

This phenomenon may be attributed to several factors. (i) Some β-lactams may bind to the LDTs/PBPs as small fragment-like compounds, engaging a single site on the protein surface through multiple binding modes with comparable affinities (36). In some cases, the binding of a small molecule might induce both stabilizing and destabilizing effects on different regions of the protein, which could compensate for each other, resulting in no net change in the melting temperature (37). (ii) The binding site of the β-lactams on the protein might not be critical for the overall thermal stability. If β-lactam binds to a region that does not significantly affect the protein’s folding or structural integrity, it may not cause a noticeable change in the melting temperature.

In summary, this study identified a highly efficacious combination of durlobactam + imipenem which leveraged a dual synergy mechanism against *Mab*. Specifically, this mechanism-informed BL/BLI combination was the most efficacious regimen due to its ability to target multiple LDTs and PBPs (target redundancy) while inhibiting Bla_Mab_. Clinical data suggest potential benefits of combining β-lactams to achieve target redundancy (38–41). For chronic infections such as *Mab*, bactericidal activity is likely more crucial than merely inhibiting bacterial growth. Although synergy is observed in MIC measurements, the predictive value is likely greater for the synergy and bactericidal activity in *in vitro* time-kill studies. Further evaluation through dynamic *in vitro* and animal infection models, as well as clinical trials, is required to validate these findings and optimize treatment strategies. Overall, these findings enhance our understanding of the binding dynamics of β-lactams and BLIs to LDTs and PBPs, providing valuable insights into their potential effectiveness in clinical applications.

## MATERIALS AND METHODS

### Bacterial strains, antibiotics and reagents

We studied *Mab* strain (ATCC 19977) which produces Bla_Mab_. This strain was used for MIC testing, static drug-concentration time-kill experiments (SCTK), as well as morphological changes with flow cytometry and confocal microscopy. The active compounds of cefuroxime salts, imipenem, amoxicillin, sulbactam, and avibactam were purchased from AchemBlock. Sulopenem was sourced from Iterum Therapeutics, and durlobactam was provided from Innoviva Specialty Therapeutics. All β-lactams and BLIs were prepared in sterile distilled water and subsequently filter-sterilized using a 0.22 µm polyethersulfone syringe filter.

### The MIC testing

The MICs were determined in duplicate according to the Clinical Laboratory Standards Institute (CLSI) guidelines (42) using Middlebrook 7H9 broth supplemented with 10% (vol/vol) oleic acid-albumin-dextrose-catalase (OADC) and 0.05% (vol/vol) Tween 80, with an inoculum of 5 × 10^5^ CFU/mL. MIC testing was conducted with six β-lactams in the presence and absence of BLIs, either avibactam or durlobactam + sulbactam. Avibactam was added at a fixed concentration of 4 mg/L to serial dilutions of β-lactams, while durlobactam + sulbactam was tested by adding a fixed concentration of 1 mg/L to serial dilutions of β-lactams or combined with β-lactams at a 1:1 ratio. *Mab* isolates were incubated with the test antibiotics at 30°C for 48 h. The MIC was defined as the lowest antibiotic concentration that prevented visible bacterial growth.

### SCTK assay

The SCTK studies were performed over 10 days in Middlebrook 7H9 broth, supplemented with 10% (vol/vol) OADC, 0.2% (vol/vol) glycerol, and 0.05% (vol/vol) Tween 80, in duplicate. The efficacy of β-lactams and BLIs was assessed using ATCC 19977 at an initial inoculum of 10^5.5^ to 10^6.3^ CFU/mL. The concentrations of β-lactams and BLIs were selected based on MIC values and clinically relevant levels, informed by the predicted average unbound steady-state concentrations (*f*C_ss,avg_) in plasma at clinically relevant daily doses (Table 3, supplement, and Sayed et al. (19)). The concentrations of each drug used in this assay were as follows: imipenem at 12 mg/L, ceftaroline at 8 mg/L, cefuroxime at 8 mg/L, amoxicillin at 2 mg/L, sulopenem at 2 mg/L, tebipenem at 1.5 mg/L, amikacin at 12 mg/L, clarithromycin at 0.3 mg/L, and cefoxitin at 7 mg/L. To mitigate thermal degradation, the following supplementation was performed every 48 h: 75% for imipenem, 33% for sulopenem, 54% for tebipenem, 50% for cefuroxime, and 55% for cefoxitin, based on β-lactam stability data (43, 44). The broth was replaced with fresh broth containing the appropriate drug concentrations every 3 days. In brief, the bacterial suspension was centrifuged at 4,500 × *g*, and the supernatant containing the antibiotics was discarded. Fresh broth with antibiotics was then added accordingly. For viable counting, 100 µL of either undiluted or appropriately diluted samples was subcultured on Middlebrook 7H10 agar plates supplemented with 1% (vol/vol) OADC, 0.2% glycerol, and 0.05% (vol/vol) Tween 80. All samples were washed twice with sterile 0.9% saline before serial dilutions or plating onto the agar plates

### Cloning and purification of LDTs and PBPs

Truncated sequences of LDT1-5, DDC, PBP B, and PBP-lipo (Δ1–41) were generated by Celtek Biosciences and cloned into the pET28(a) + vector with a tobacco etch virus (TEV) protease cleavage site before the start codon. Clones were transformed into Escherichia coli BL21 (DE3) and grown to an OD600 of 0.6–0.8. Protein expression was induced with 0.25 mM isopropyl β-d-1-thiogalactopyranoside (IPTG) and incubated for 18 h at 18°C. Cell pellets were collected and stored at −20°C overnight, then resuspended in a buffer containing 50 mM Tris (pH 8.0), 400 mM sodium chloride, and 1 mM Tris (2-carboxyethyl) phosphine hydrochloride (TCEP), followed by sonication and centrifugation. The supernatant was purified using a His Prep FF 16/10 column (GE Healthcare) and eluted with 500 mM imidazole. Eluted protein was dialyzed overnight at 4°C in a buffer with 50 mM Tris (pH 8.0), 150 mM sodium chloride, and 0.5 mM TCEP in the presence of His-tagged TEV protease. To remove the His tag, the mixture was passed over the His Prep FF 16/10 column again. PBP-lipo and LDT5 did not undergo His-tag removal with TEV protease due to instability issues.

### Mass spectrometry analysis

The purified proteins listed above were incubated at room temperature with β-lactams or BLIs at a molar ratio of 1:20 for 2 h. The reactions were then quenched with acetonitrile and diluted to 1 mL with 0.1% formic acid in water. Samples were analyzed using a quadrupole time-of-flight (Q-TOF) Waters Synapt-G2-Si electrospray ionization mass spectrometer (ESI-MS) and a Waters Acquity H class ultra-performance liquid chromatography (UPLC) system, equipped with a BEH C18 1.7 µm column (2.1 × 50 mm). The MS was tuned with the following settings: capillary voltage set at 3 kV, sampling cone at 35 V, source offset at 35, source temperature of 100°C, desolvation temperature of 500°C, cone gas flow rate at 100 L/h, desolvation gas flow rate at 800 L/h, and nebulizer set at 6.0. The mobile phases consisted of 0.1% formic acid in water and 0.1% formic acid in 100% acetonitrile, with gradient elution from 10% to 90% of 0.1% formic acid in water over 11 min. The system’s mass accuracy was ±5 Da.

### Bocillin-FL binding assay

To analyze the binding of PBP-lipo with β-lactams or BLIs, purified PBP-lipo (4 µg) was incubated with β-lactams or BLIs at a 1:10 molar ratio in a buffer containing 10 mM sodium phosphate (pH 7.4) and 150 mM NaCl. The incubation was carried out for 2 h at room temperature. After the initial incubation, 1 µM Bocillin FL was added to the reaction mixture. The incubation was continued for an additional 3–4 h at 37°C. After incubation, the samples were boiled to denature the proteins. The samples were then subjected to SDS-PAGE. The PBP-lipo:Bocillin complexes were visualized by fluorescence analysis at a wavelength of 488 nm using an Azure 300 gel imaging system. The intensity of the PBP-lipo:Bocillin complex bands was analyzed using GelAnalyzer software. The intensity of the band for PBP-lipo alone (without β-lactams) was set to 1 as the highest intensity. The intensity of each band for PBP-lipo incubated with β-lactams was then normalized by dividing by the intensity of the band for PBP-lipo alone.

### Kinetics

Steady-state kinetic studies were conducted using purified LDTs and PBPs (LDT1-5, PBP B, PBP-lipo, and DDC) as previously described (20). The assays were performed using a BioTek Synergy2 multimode reader in conjunction with Gen5 analysis software. Increasing concentrations of NCF (Δε_482_ = 17,400 M^⁻¹^ cm^⁻¹^) were used, while fixed concentrations of the LDTs and PBPs were monitored for changes in absorbance at 482 nm over a time period of 0 to 10 min. To determine reaction velocities, a 0.25 cm path length was employed. The steady-state kinetic parameters (V_max_, k_cat_, and K_m_) were obtained by fitting the data to the Henri-Michaelis-Menten equation using a nonlinear least-squares fit in Origin 8.1 software (OriginLab, Northampton, MA). The reaction rate (v) at a given substrate concentration ([S]) was calculated according to the following equations:


$$v=\frac{V_{max}\times [S]}{K_{m}+[S]}$$

$$k_{cat}=V_{max}/\left[E\right]$$

To determine the apparent inhibition constants (*K_i,app_*) for β-lactams and BLIs, the following experimental condition was employed: NCF as a substrate at a concentration of 5 × Km, LDT/ PBP enzymes with fixed concentration, and β-lactams and BLIs with various concentrations. Changes in absorbance at 482 nm were measured over a period of 0–30 min for each concentration of β-lactams and BLIs. Data were linearized using a Dixon plot, plotting the inverse changes in absorbance (1/ΔA) against the concentrations of β-lactams and BLIs. The observed *K_i,app_* was determined directly from the Dixon plot, and these values were corrected for substrate concentration and affinity for the LDT using the following equation:


$$K_{i,app}=\frac{observed\;K_{i,app}}{1+\left[NCF\right]/K_{m}^{\;\;\;NCF}}$$

### Differential scanning fluorimetry analysis

Each purified protein (2–4 µM) was incubated with or without β-lactams or BLIs at a molar ratio of 1:50 at room temperature for 2 h. Following incubation, SYPRO Orange dye (10× concentration; Fisher Scientific) was added. The samples were then brought to a final volume of 30 µL with PBS buffer and subjected to thermal cycling in a qPCR machine (Bio-Rad), with the temperature increasing from 25°C to 95°C at a rate of 1°C/min. Fluorescence changes were monitored with an excitation wavelength of 492 nm. The experiments were conducted at least in duplicate, and the melting temperature (*T_m_*) was determined for each sample. The *T_m_* values were then compared to those of the protein in the absence of β-lactams or BLIs to ascertain the change in melting temperature (Δ*T_m_*).

### Circular dichroism spectroscopy

The circular dichroism spectra of LDT2 (5 µM in PBS buffer) were recorded on a circular dichroism spectrometer with a 2 mm path length, both in the absence and presence of 10 µM imipenem, ceftaroline, sulopenem, and cefuroxime at 25°C. To ensure the detection of conformational changes, LDT2 and the β-lactams were incubated at room temperature for 2 h. The contribution of each β-lactam alone was subtracted in each case.

### Flow cytometry

Flow cytometry was employed to evaluate morphological changes in individual bacterial cells following PBP-lipo inactivation after 24 h of β-lactam treatment. The bacterial cells were stained using the BacLight kit L7012 (Thermo Fisher Scientific, Waltham, MA), containing SYTO 9 and propidium iodide (PI). Analysis was performed using a CytoFLEX flow cytometer (Beckman Coulter, CA), with data processed using CytExpert software.

### Confocal microscopy

Bacterial morphological changes resulting from PBP-lipo inactivation were investigated using an ImageXpress Micro Confocal Microscope (Molecular Devices, CA). The staining procedures applied to the samples mirrored those used for flow cytometry. Stained bacterial cells were placed in optically clear flat-bottom 96-well plates, and observations were conducted at 60× magnification. Cell length measurements were obtained through automated image analysis using MetaXpress 6 software.

### CART analysis

CART analysis was conducted to identify the optimal β-lactam combination for maximizing bacterial killing over 5 days. This analysis was based on static time-kill profiles, with three properties (inclusion of BLIs, use of double β-lactam combinations, and the specific type of BLIs) as independent variables and bacterial burden as the dependent variable. To evaluate model performance, the data set was split into training (70%) and testing (30%) subsets. The training data were used to identify the best predictor values during the construction of the CART model. Tenfold cross-validation was employed to prevent overfitting. The Receiver Operating Characteristic (ROC) curve was utilized to select the optimal CART model, focusing on the nested subtree with the smallest misclassification cost. The CART analysis was performed using Minitab software (version 21.4.2, Minitab, LLC, State College, PA).

## SUPPLEMENTAL MATERIAL

Supplemental material aac.01174-24-s0001.docx Additional methods; Figures S1 to S3.

ASM does not own the copyrights to Supplemental Material that may be linked to, or accessed through, an article. The authors have granted ASM a non-exclusive, world-wide license to publish the Supplemental Material files. Please contact the corresponding author directly for reuse.
