# Supplementary material for: Durlobactam in combination with β-lactams to combat Mycobacterium abscessus
Source: Antimicrob Agents Chemother. 2024 Dec 23;69(2):e01174-24. doi: 10.1128/aac.01174-24 (PMC11823594; doi:10.1128/aac.01174-24)
Supplement: Supplemental material — Additional methods; Figures S1 to S3. [file aac.01174-24-s0001.docx]

**Supplement**

**Predicted average unbound steady-state concentrations (*f*C_ss,avg_) of cefuroxime, amikacin, and clarithromycin at clinical relevant doses given as continuous infusion.**

The total clearance of cefuroxime after IV dosing has been reported to range from approximately 9.0 to 16 L/h in healthy volunteers (with normal renal function) and critically-ill patients.[^1-3^](#_ENREF_1) And cefuroxime is 33 to 50% bound in human plasma.[^4^](#_ENREF_4) At a dose of 1500 mg IV cefuroxime every 8 h, these clearances result in unbound average drug concentrations at steady-state between 6.6 and 12 mg/L. Therefore, we studied cefuroxime concentration of 8 mg/L, which fell within this range. In contrast, oral dosing of 500 mg cefuroxime axetil every 8 h would result in an average unbound steady-state concentration of approximately 1.7 mg/L.[^4^](#_ENREF_4)

We studied 12 mg/L amikacin as an average unbound concentration at steady-state for amikacin doses between 20 and 30 mg/kg. This concentration fell well within the range of amikacin concentrations observed in critically ill patients.[^5-7^](#_ENREF_5)

We studied time kill study with 0.3 mg/L of clarithromycin as the steady-state average unbound concentration for a daily dose of 1000 mg. This concentration is consistent with the range of amikacin levels typically observed in patients.[^8^](#_ENREF_8)

**Figure S1:** Fluorescence imaging of SDS-PAGE gel showing *Mab* PBP-lipo and *Mab* PBP B after reaction with Bocillin™-FL. After a 30-minute incubation (left panel), the PBP-lipo-Bocillin™ complex band was not observed. Only after 3 hours or longer did *Mab* PBP-lipo bind to Bocillin™, allowing the detection of the PBP-lipo-Bocillin™ complex (right panel).


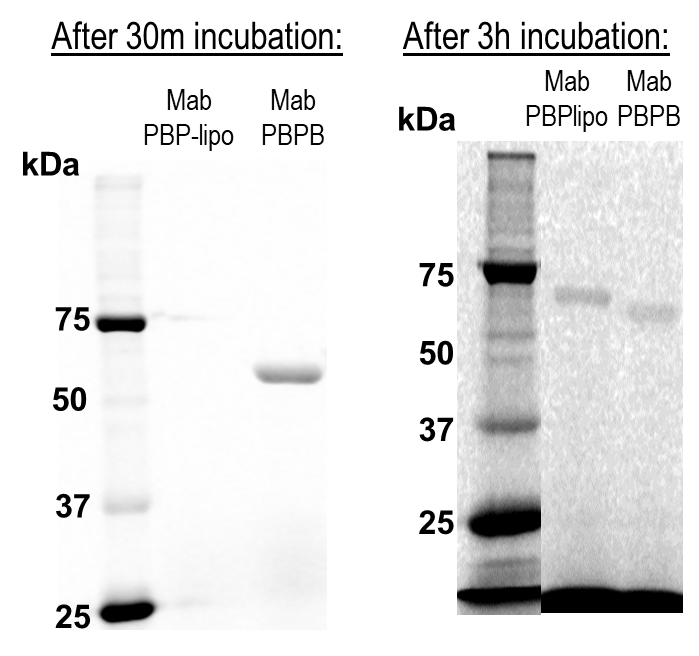


**Figure S2:** Thermal shift assay using Differential Scanning Fluorimetry (DSF), conducted to probe changes in protein stabilization of *Mab* LDT1-5, DDC, PBP B, and PBP-lipo in response to binding to various β-lactams and BLIs. The rate of change in SYPRO Orange fluorescence was plotted against temperature for proteins in the presence and absence of β-lactams and BLIs. The melting temperatures (*T_m_*) were measured at least in duplicate and are presented in Table 4.


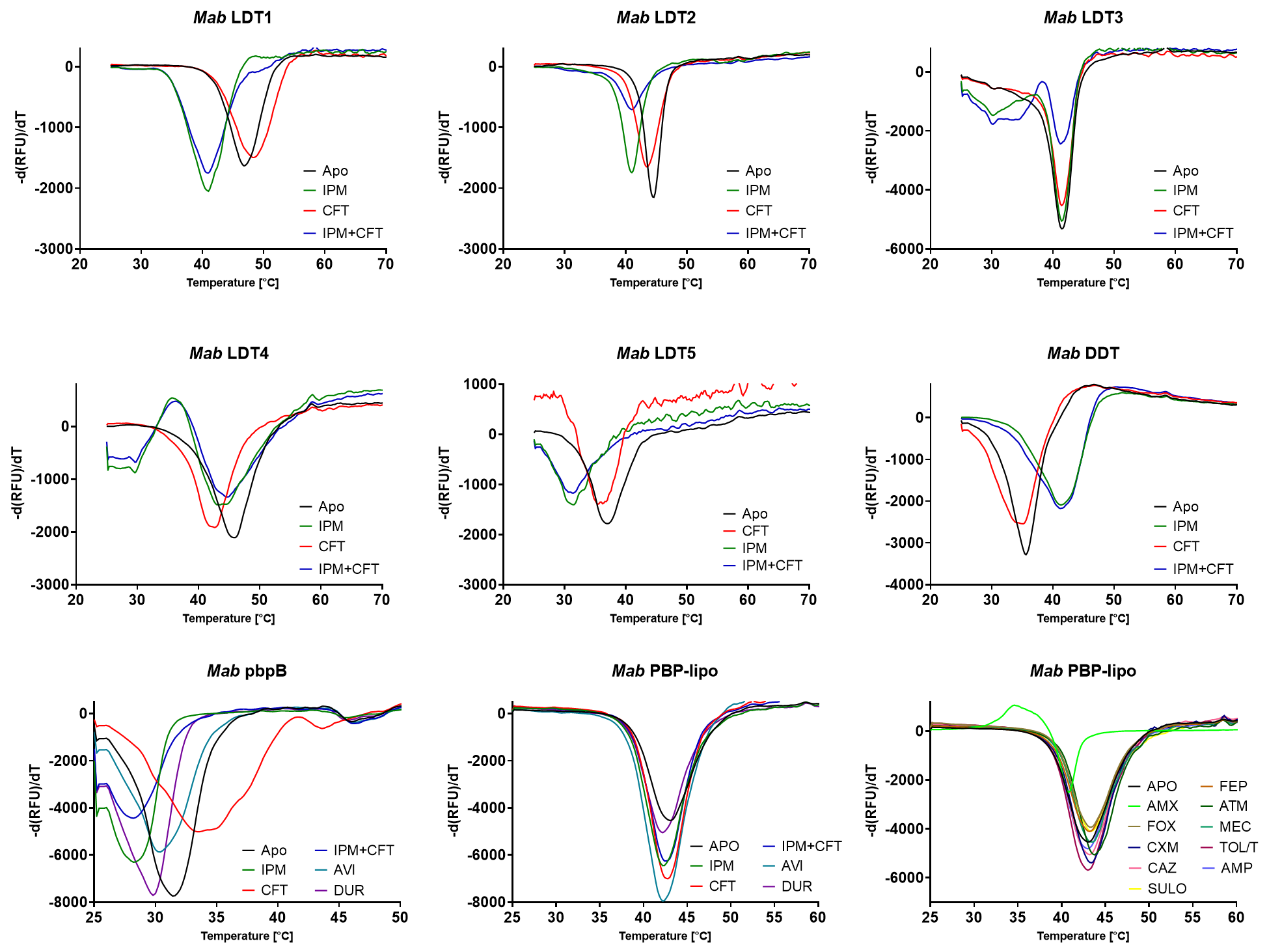


**Figure S3:** CD spectroscopy assay demonstrating conformational changes of *Mab* LDT2 protein upon binding with ceftaroline (CFT), imipenem (IPM), their combination (IPM+CFT), sulopenem (SULO), and cefuroxime (CXM). CD spectra were recorded at wavelengths of 200–250 nm. The CD spectrum of LDT2 exhibited changes in the 208–220 nm range upon binding with each β-lactam, indicating potential conformational changes after binding.


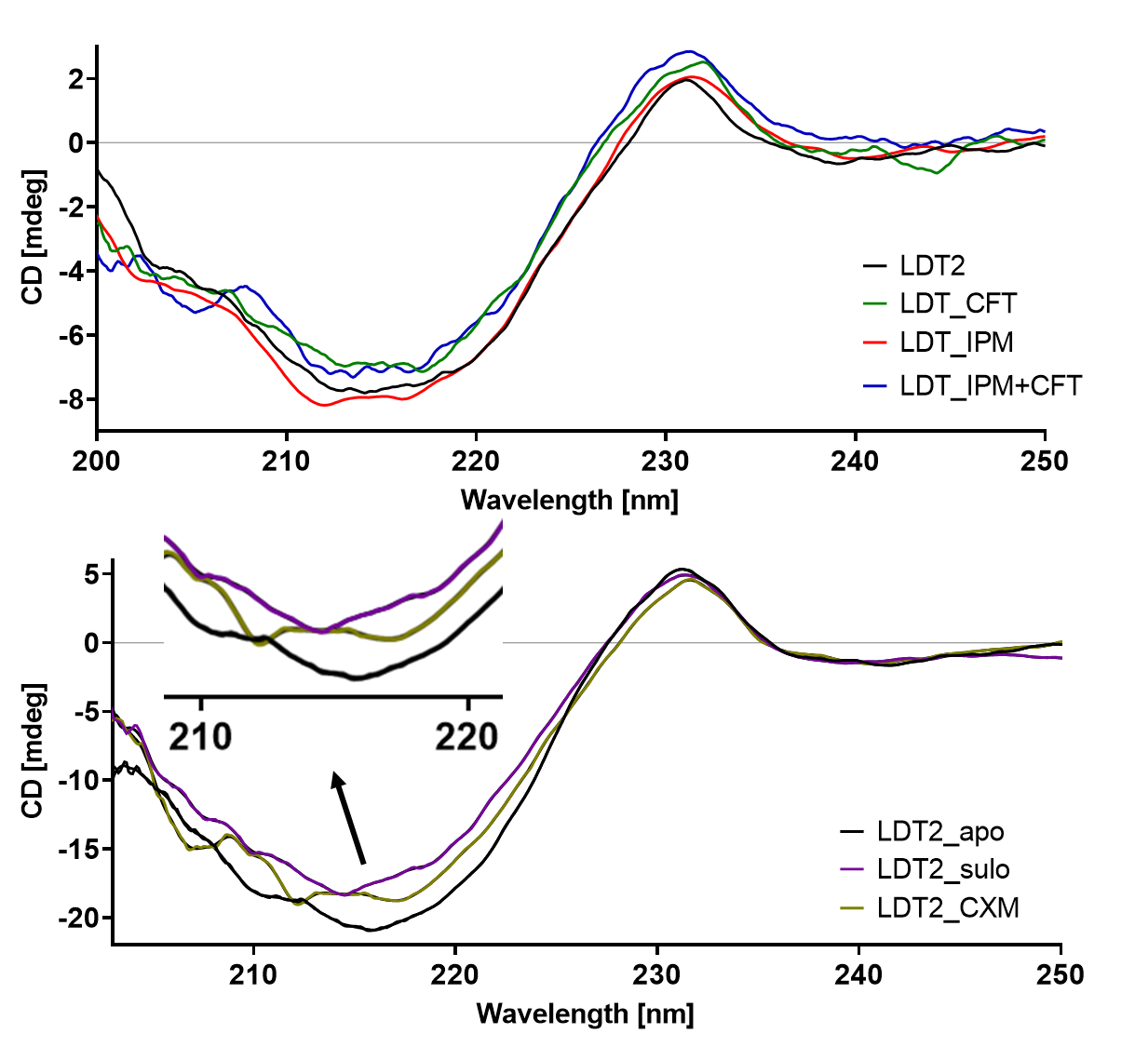


**Supplement References**

1. **Thonnings S, Jensen KS, Nielsen NB, Skjonnemand M, Hansen DS, Lange KHW, and Frimodt-Moller N.** Cefuroxime pharmacokinetics and pharmacodynamics for intravenous dosage regimens with 750 mg or 1500 mg doses in healthy young volunteers. Journal of Medical Microbiology **2020**. 69**:**387-395.

2. **Carlier M, Noë M, Roberts JA, Stove V, Verstraete AG, Lipman J, and De Waele JJ.** Population pharmacokinetics and dosing simulations of cefuroxime in critically ill patients: non-standard dosing approaches are required to achieve therapeutic exposures. Journal of Antimicrobial Chemotherapy **2014**. 69**:**2797-2803.

3. **Foord RD.** Cefuroxime - Human Pharmacokinetics. Antimicrobial Agents and Chemotherapy **1976**. 9**:**741-747.

4. **Bulitta JB, Landersdorfer CB, Kinzig M, Holzgrabe U, and Sorgel F.** New Semiphysiological Absorption Model To Assess the Pharmacodynamic Profile of Cefuroxime Axetil Using Nonparametric and Parametric Population Pharmacokinetics. Antimicrobial Agents and Chemotherapy **2009**. 53**:**3462-3471.

5. **Burdet C, Pajot O, Couffignal C, Armand-Lefèvre L, Foucrier A, Laouènan C, Wolff M, Massias L, and Mentrè F.** Population pharmacokinetics of single-dose amikacin in critically ill patients with suspected ventilator-associated pneumonia. European Journal of Clinical Pharmacology **2015**. 71**:**75-83.

6. **Delattre IK, Musuamba FT, Nyberg J, Taccone FS, Laterre PF, Verbeeck RK, Jacobs F, and Wallemacq PE.** Population Pharmacokinetic Modeling and Optimal Sampling Strategy for Bayesian Estimation of Amikacin Exposure in Critically Ill Septic Patients. Therapeutic Drug Monitoring **2010**. 32**:**749-756.

7. **Jiao YY, Yan J, Sutaria DS, Lu P, Vicchiarelli M, Reyna Z, Ruiz-Delgado J, Burk E, Moon E, Shah NR, Spellberg B, Bonomo RA, Drusano GL, Louie A, Luna BM, and Bulitta JB.** Population pharmacokinetics and humanized dosage regimens matching the peak, area, trough, and range of amikacin plasma concentrations in immune-competent murine bloodstream and lung infection models. Antimicrobial Agents and Chemotherapy **2024**. 68.

8. **Abduljalil K, Kinzig M, Bulitta J, Horkovics-Kovats S, Sörgel F, Rodamer M, and Fuhr U.** Modeling the Autoinhibition of Clarithromycin Metabolism during Repeated Oral Administration. Antimicrobial Agents and Chemotherapy **2009**. 53**:**2892-2901.
